# Identification of plasma microRNAs as a biomarker of sporadic Amyotrophic Lateral Sclerosis

**DOI:** 10.1186/s13041-015-0161-7

**Published:** 2015-10-24

**Authors:** Ikuko Takahashi, Yuka Hama, Masaaki Matsushima, Makoto Hirotani, Takahiro Kano, Hideki Hohzen, Ichiro Yabe, Jun Utsumi, Hidenao Sasaki

**Affiliations:** Department of Neurology, Hokkaido University Graduate School of Medicine, North 15 West 7, Kita-ku, Sapporo, Hokkaido 060-8638 Japan; Department of Neurology, Obihiro Kosei General Hospital, West 6, South 8, Obihiro, Hokkaido 080-0016 Japan; Cancer Institute, Japanese Foundation for Cancer Research, 3-8-31, Ariake, Koto, Tokyo, 135-8550 Japan

**Keywords:** Amyotrophic lateral sclerosis, microRNA, Biomarker

## Abstract

**Background:**

Amyotrophic Lateral Sclerosis (ALS) is a fatal neurodegenerative disease, which leads to the loss of upper and lower motor neurons, with a currently unknown etiology. Specific biomarkers could help in early detection and diagnosis, and could also act as indicators of disease progression and therapy effectiveness. MicroRNAs (miRNAs) are small (18–25 nucleotides), single-stranded non-coding RNA molecules that play important regulatory roles in animals and plants by targeting mRNAs for cleavage or translational repression, and are essential for nervous system development. Many of the genes associated with genetic ALS have pathological biological pathways related to RNA metabolism, and their pathogenesis may be affecting the maturing processes of miRNA.

**Results:**

We compared miRNA from the plasma of sALS patients and healthy controls using two cohorts; a discovery cohort analyzed with microarray (16 sALS patients and ten healthy controls) and a validation cohort confirmed with qPCR (48 sALS patients, 47 healthy controls and 30 disease controls). We measured the total amount of extracted RNA along with a spike-in control that ensured the quality of our quantification. A percentage of the 10–40 nt RNAs extracted from the total RNA showed a significant increase in ALS patients. There was a negative correlation between total RNA concentration and disease duration from onset to end point. Three of the miRNAs were up-regulated and six were down-regulated significantly in the discovery cohort. Since an internal control is required as a sample stability indicator of both the patients and controls in microarray analysis, we selected the miRNA showing the smallest dispersion and equivalency between the two groups’ mean value, and decided to use hsa-miR-4516. We found hsa-miR-4649-5p to be up-regulated, and hsa-miR-4299 to be down-regulated, where each was not influenced by clinical characteristics. *EPHA4*, a target gene linked to the nervous system which has also been reported to be a disease modifier of ALS, is the common and most notable target gene of hsa-miR-4649-5p and hsa-miR-4299.

**Conclusion:**

We have shown the relationship circulating plasma miRNA has with both healthy controls and diseased patients. Hsa-miR-4649-5p and hsa-miR-4299 have the potential to be ALS diagnosis biomarkers.

**Electronic supplementary material:**

The online version of this article (doi:10.1186/s13041-015-0161-7) contains supplementary material, which is available to authorized users.

## Background

Amyotrophic Lateral Sclerosis (ALS) is a fatal neurodegenerative disease which leads to the loss of upper and lower motor neurons, with a currently unknown pathogenesis. Specific biomarkers could help in early detection and diagnosis, and could also act as indicators of disease progression and therapy effectiveness. Numerous biomarker candidates have emerged, but none have been directly linked to the cause of ALS.

MicroRNAs (miRNAs) are small (18–25 nucleotides), single-stranded, non-coding RNA molecules that play important regulatory roles in animals and plants by targeting mRNAs for cleavage or translational repression [1]. Each miRNA is estimated to regulate around 200 targets, and mRNA transcripts may be regulated by multiple miRNAs [2, 3]. RNA polymerase II transcribes pri-miRNA from the miRNA gene, and Drosha, RNase III enzyme and its binding partner DGCR8 process pri-miRNA into hairpin structures called pre-miRNA [4]. The pre-miRNAs are then exported by Exportin 5 into the cytoplasm, where a second RNase III enzyme Dicer cleaves the pre-miRNA to generate double-stranded miRNA duplexes. Further, these duplexes are bound with Argonaute 2 (AGO2), an important component of the RNA-induced silencing complex, and are selected to be mature miRNAs [5]. MiRNAs play an important role in nervous system development [6, 7]. Haramati et al. found that disturbing the miRNA biogenesis pathway by deleting Dicer from spinal motor neurons in mice caused a Spinal Muscular Atrophy (SMA) like phenotype [8]. MiRNA expression and its role in neuronal diseases including amyotrophic lateral sclerosis have been suggested, but specifics are yet to be understood [9–14].

MiRNAs are found in biological fluids such as blood, urine and cerebrospinal fluid (CSF) and are said to be stable because they are encapsulated in exosomes or found in complexes with Argonaute proteins and lipoproteins which make them resistant to RNase present in the circulating environment [15–17]. In our study, we compared miRNA from the plasma of sALS patients with healthy controls using two cohorts; a discovery cohort analyzed with microarray and a validation cohort confirmed with qPCR, to ensure the repeatability of the quantitative analysis.

## Results

### Patient characteristics

There were 16 sALS patients and ten healthy controls in the miRNA microarray discovery cohort, and 48 patients and 77 controls (47 healthy and 30 disease (Parkinson’s)) in the reverse transcription-quantitative polymerase chain reaction (RT-qPCR) validation cohort. The number of subjects and patient’s characteristics are provided in Table 1. The patients and controls of each cohort did not overlap. Although there were a variety of individuals in the discovery cohort, there were no significant gender or age biases between the groups in the validation cohort. Seven sALS patients of the validation cohort provided their blood two or more times (four at most), and samples were taken at six or 12 month intervals. The samples collected a second time and later were used only for time duration studies which are described later and were not included in the validation cohort.

**Table 1:** General and clinical characteristics of the study subjects respective of cohort

Type of cohort		Discovery cohort (*N* = 26)		Validation cohort (*N* = 95)		
Patients’ characteristics		sALS patients (*n* = 16)	Controls (*n* = 10)	sALS patients (*n* = 48)	Healthy controls (*n* = 47)	Disease controls (*n* = 30)
Age (mean ± SD)		65.62 ± 9.11	49.30 ± 14.88	66.67 ± 10.52	67.08 ± 10.89	69.00 ± 5.91
Gender (n)	Male	9	5	29	29	17
	Females	7	5	19	18	13
Disease duration from onset to collection, months (mean ± SD)		17.18 ± 9.29		20.04 ± 18.43		
Initial symptoms (n)	Bulbar	3		20		
	Upper limb	10		17		
	Lower limb	3		11		
ALSFRS-R (mean ± SD)		36.06 ± 10.59		33.15 ± 11.72		
Bulbar paralysis scores of ALSFRS-R (mean ± SD)		9,75 ± 3.51		8.06 ± 4.04		
Barthel Index (mean ± SD)		72.19 ± 31.99		68.02 ± 34.23		
Disease duration from onset to end point, months (mean ± SD)		31.00 ± 13.66		28.58 ± 18.72		

### Quantification and analysis of the total RNA extracted from plasma

**Fig. 1:**
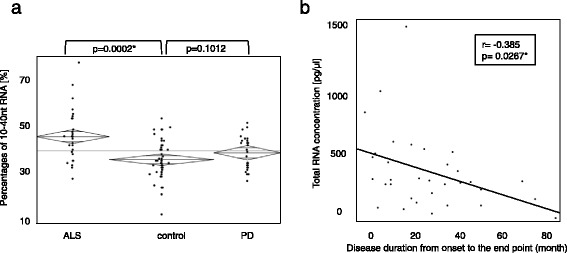
The concentration of extracted plasma RNA. **a** Percentages of 10-40 nt RNA including miRNA that were significantly increased in ALS (*p* = 0.0002*). **b** A scatterplot of disease duration from onset to end point and total RNA concentration, showing a negative correlation (r = −0.385, *p* = 0.0267*)

The RNA extracted from the validation cohort was immediately quantified using a Bioanalyzer 2100. Among the miRNA of sALS patients, only the very small RNA (10–40 nt) group had a significant increase (Fig. 1a). In a multivariable analysis that included clinical characteristics, the total RNA concentration and disease duration from onset to end point showed a negative correlation (r = −0.385, *p* = 0.0267*) (Fig. 1b).

### Differential expression of plasma miRNA in ALS patients compared with healthy controls using microarray

Using the 3D-Gene microarray system, we were able to quantify 1800 total human miRNAs in the discovery cohort. Three of the sufficiently detected miRNAs were up-regulated in sALS patients’ plasma (Table 2). 120 of the miRNAs were significantly down-regulated in ALS patients. We selected only the top 6 miRNAs showing a fold change of less than 0.5, but also, sufficient expression in healthy controls’ plasma (hsa-miR-26b-5p, hsa-miR-4299, hsa-let-7f-5p, hsa-miR-4419a, hsa-miR-3187-5p and hsa-miR-4496) (Table 2).

**Table 2:** Up- or down- regulated miRNAs using microarray analysis and extraction of an internal control

Differential expression type	MiRNA name	Fold change	*p* value	
			Student *t*-test	Wilcoxon sum ranked test
Up-regulated	hsa-miR-4258	1.109	<0.0001*	0.0001*
	hsa-miR-663b	1.117	0.0004*	0.0017*
	hsa-miR-4649-5p	1.045	0.0659	0.179
Down-regulated	hsa-miR-26b-5p	0.374	<0.0001*	0.0001*
	hsa-miR-4299	0.435	0.0001*	0.0006*
	hsa-let-7f-5p	0.464	<0.0001*	0.0002*
	hsa-miR-4419a	0.464	0.0001*	0.0002*
	hsa-miR-3187-5p	0.485	0.0002*	0.0001*
	hsa-miR-4496	0.495	0.0003*	0.0011*
Candidates of Internal control	hsa-miR-4516	0.999	0.9340	

*p < 0.05

The miRNAs that were found to be significantly expressed were multivariable-analyzed along with clinical characteristics. Up-regulated miRNAs hsa-miR-663b and hsa-miR-4649-5p showed a negative correlation with disease duration from onset to end point (hsa-miR-663b; r = −0.612, *p* = 0.0263*, hsa-miR-4649-5p; r = −0.732, *p* = 0.0045*)

Since in RT-qPCR analysis an internal control is required as a sample and PCR stability indicator, we selected the miRNA showing the smallest dispersion and equivalency between the two groups’ mean value, and decided to use hsa-miR-4516.

### Validation analysis using RT-qPCR

To ensure sample quality and sufficient extraction amounts, we calculated the return rate with a spike-in control (cel-miR-39-3p), which was found sufficiently in every sample. Hsa-miR-4516 was the most stable when relative quantification normalized with an internal control was used, so we decided to use this as the internal control.

**Fig. 2:**
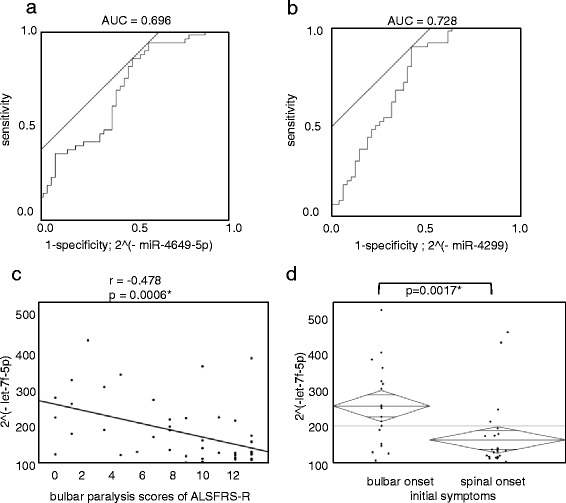
The miRNAs significantly expressed in the validation cohort. **a** The ROC curve of hsa-miR-4649-5p and ALS/control, where the AUC value was 0.696. **b** The ROC curve of hsa-miR-4299 and ALS/control, where the AUC value was 0.728. **c** The negative correlation of hsa-let-7f-5p with bulbar paralysis scores of ALSFRS-R. **d** Showing hsa-let-7f-5p as decreasing more in spinal-onset patients than in non-spinal-onset patients

Hsa-miR-4649-5p was confirmed as being significantly up-regulated (relative expression; 1.532, *p* = 0.0009*) in the plasma of ALS patients. There were no significant differences between ALS patients and normal controls for hsa-miR-4258 and hsa-miR-663b (Table 3). Hsa-miR-4299 was significantly down-regulated in the plasma of ALS patients in both of the normalization methods (relative expression; 0.026, *p* = 0.0001*), Hsa-miR-26b-5p did not have a significant expression change. Unfortunately, the analysis of three miRNAs (hsa-miR-4419a, hsa-miR-3187-5p, hsa-miR-4496) failed because they formed nonspecific primer dimers. A comparison of each parameter with the Receiver Operating Characteristic (ROC) curve showed that hsa-miR-4649-5p and hsa-miR-4299 showed a high area under the curve (AUC) value (hsa-miR-4649-5p; AUC = 0.696, hsa-miR-4299; AUC = 0.728) (Fig. 2a, b). Hsa-miR-4649-5p and hsa-miR-4299 didn’t show any significant differences with Parkinson’s disease (PD) patients and controls, but hsa-miR-663b was found to be up-regulated in PD patients (relative expression; 1.328, *p* = 0.0094*) (Table 4).

**Table 3:** The comparison of up- or down- regulated miRNAs between ALS and healthy controls using qPCR

differential expression type	MiRNA name	Relative expression^a^	p value
Up-regulated	hsa-miR-4258	0.990	0.4605
	hsa-miR-663b	1.090	0.9935
	hsa-miR-4649-5p	1.532	0.0009*
Down-regulated	hsa-miR-26b-5p	1.095	0.6574
	hsa-miR-4299	0.026	0.0001*
	hsa-let-7f-5p	1.105	0.6508

*p < 0.05

^a^Average of the expression level of internal control (hsa-miR-4516) = 1

**Table 4:** The comparison of up- or down- regulated miRNAs between PD and healthy controls using qPCR

differential expression type	MiRNA name	Relative expression^a^	p value
Up-regulated	hsa-miR-4258	0.753	0.1197
	hsa-miR-663b	1.328	0.0094*
	hsa-miR-4649-5p	0.927	0.2498
Down-regulated	hsa-miR-26b-5p	0.917	0.0800
	hsa-miR-4299	1.950	0.1032
	hsa-let-7f-5p	0.603	0.2470

*p < 0.05

^a^Average of the expression level of internal control (hsa-miR-4516) = 1

When miRNAs were analyzed against clinical characteristics, hsa-let-7f-5p and bulbar paralysis scores of the ALS Functional Rating Scale revised (ALSFRS-R) showed a negative correlation (r = −0.478, *p* = 0.0006* (Fig. 2c). Hsa-let-7f-5p was found to be especially decreased in patients whose initial symptoms appeared as spinal-onset (*p* = 0.0017*)(Fig. 2d). The other miRNAs seemed to not be influenced by clinical characteristics. Hsa-miR-4649-5p did not show a negative correlation with disease duration from onset to end point.

### Hsa-miR-663b increased over time in individual patients

**Fig. 3:**
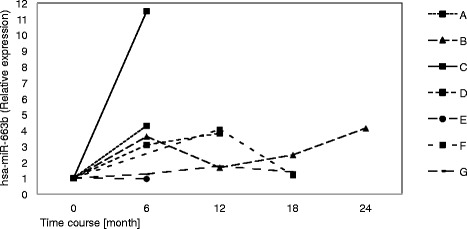
Hsa-miR-663b increased in the time course. The seven patients we were able to follow over time, and hsa-miR-663b showing an increase over time. Relative expressions are shown as the first collection standard

A follow-up collection was possible with seven of the patients in the validation cohort, where the longest following of a patient was 24 months. There wasn’t a significant difference in hsa-miR-663b when comparing sALS patients and healthy controls, but there was a tendency to increase over time for this miRNA (Fig. 3). We confirmed the magnitude of increase with copy numbers, calculated using total RNA concentration and a spike-in-control (cel-miR-39-3p) (Additional file 1). The other miRNAs did not show a clear trend.

## Discussion

We have presented a study showing the potential of circulating cell-free miRNAs to be disease biomarkers in sALS, using two different methods. Significantly expressed miRNAs in a discovery cohort using microarray analysis were confirmed as being either up- or down-regulated with a validation cohort using RT-qPCR, a methodology completely different from microarray.

When determining the quantity of miRNA in a circulating environment, there are several points to consider. Firstly the housekeeping gene is not fixed unlike those in the intercellular environment. Secondly, because of the small molecular weight of the miRNA, a comparison with a spike-in control requires the concentration of the control be adjusted accordingly. Finally, to do a simple comparison between a miRNA and a spike-in-control, it is understood that the miRNA amounts in each are equal. This is because the normalization method of microarray is based on the assumption that concentrations of the subjects are equal. Microarray systems usually analyze whole species of miRNA, hence with thinner miRNA which were close to the lower limit of luminance detection, their up- or down-regulation may not be satisfactorily detected. We think that the miRNAs showing a common expression in both cohorts are the most suitable biomarker candidates. Quantifying the total extracted RNA and spike-in-control (cel-miR-39-3p) gave us confidence in our sample quality and reassured us of our quantification method. RT-qPCR results were analyzed using relative quantification with an internal control. We used hsa-miR-4516 as the internal control because it had the most stable expression, smallest dispersion and equivalency between the two groups’ mean value.

In the comprehensive microarray analysis, up-regulated miRNAs were rare but miRNAs that were significantly down- regulated were abundant, about 120. We narrowed our potential biomarker candidate miRNAs to the top six which showed a fold change of less than 0.5 because the amount of RNA that was extracted restricted the number of miRNAs that could be analyzed with RT-qPCR.

Many of the genes associated with genetic ALS have pathological biological processes / pathways related to RNA processing, and especially proteins belonging to hnRNP classes were reported to participate in the pathology of ALS (TARDBP and FUS) [18]. ALS pathogenesis may be affecting the synthesis and maturing processes of miRNA. It has been reported that TDP-43 (43-kDa TAR DNA-binding protein, *TARDBP*) cooperates with other microprocessors such as Drosha and Dicer to promote miRNA biogenesis [19]. The resulting distribution difference of the extracted RNA may be illustrating a failure of a RNA metabolism mechanism. We found that the 10-40 nt RNA group increased significantly, but only a few of the miRNAs were up-regulated. There are some hypotheses for this phenomenon; there may be unknown or de novo miRNA in the plasma of ALS patients, or there may be small, functional or non-functional RNA other than miRNA that may be being spliced and secreted abnormally resulting in inadequate RNA metabolism in ALS pathogenesis.

Previously, it was reported that hsa-miR-338-3p was up-regulated in ALS patients’ blood leukocyte, serum, CSF, and spinal cord and hsa-miR-1234-3p and hsa-miR-1825 were down- regulated in ALS patients’ serum [10, 11]. Our analysis showed that hsa-miR-338-3p was not significantly up-regulated (fold change 0.622; p-value of student’s *t* test; 0.112) and the mean value was relatively low within the discovery cohort (median of all miRNAs’ expression level; 25, hsa-miR-338-3p; 22.7). The comprehensive analysis of the previous report consisted of analyzing samples extracted from leukocytes while they verified their individual miRNAs using qPCR with samples extracted from serum. Additionaly, our analysis showed hsa-miR-1234-3p was down-regulated and hsa-miR-1825 was significantly down-regulated in ALS patients’ plasma (fold change; 0.665, p value of student’s *t* test; 0.017*), but, because we established a cut-off point for the fold change, we decided not to further analyze these miRNA.

Hsa-miR-4649-5p was up-regulated in both microarray analysis and RT-qPCR. It did not show a statistically significant difference in microarray but showed a strong negative correlation with disease duration from onset to end point in the validation cohort. We had high expectations for it to be a biomarker candidate so proceeded with the analysis of qPCR even though microarray results were not statistically significant. P values of the Student’s *t* test depend on the difference and number of the samples. Regretfully, recruiting similar aged controls and increasing the number of samples may have lead to similar results in both cohorts. This miRNA is transcribed from the 12th intron of *AEBP1*, chr7 [20], where the AEBP1 protein is estimated to function as a transcriptional repressor and play a role in adipogenesis and smooth muscle cell differentiation and is expressed in the brain. Gene Ontology (GO) analysis using MetaCore™ (https://portal.genego.com, Thomson Reuters Inc,. Carlsbad, CA) revealed that the top group of target genes for hsa-miR-4649-5p predicted by miRmap (mirmap.ezlab.org) has process networks for cytoplasmic microtubles, integrin-mediated cell-matrix adhesion and axonal guidance (Additional files 2 and 3). Their GO processes are protein assembly and biogenesis, regulation of astrocyte differentiation, neuron recognition, axonogenesis and central nervous system neuron development (Additional file 4).

Hsa-miR-663b was up-regulated in the plasma of PD patients in microarray analysis and showed an increase over time. It is present in chr2, not an intragenic miRNA, and also highly expressed in the brain. An analysis of biological systems revealed that their target genes are linked to the process networks of neurogenesis, transmission of nerve impulses and positive regulation cell proliferation (Additional files 2 and 3). Their GO process are activating or inhibiting dopamine and adrenergic receptor signaling pathways and nervous system neuron development (Additional file 4). The increase in hsa-miR-663b may suggest an acceleration of damage to the neuron in the latter stages of ALS.

Both hsa-miR-4649-5p and hsa-miR-663b showed a negative correlation with disease duration from onset to end point in microarray analysis. There was no correlation seen in qPCR analysis, but the total RNA concentration of these miRNA showed a negative correlation with disease duration from onset to end point. This may be a result of the microarray analysis methodology which requires the use of two duplicate reactions for every one sample of extracted RNA, which may inevitably contribute to the concentration of total RNA being different in the two reactions.

Hsa-let-7f-5p is one of the let-7 family members confirmed to be in a wide range of species. It is expressed highly in a lot of organs including the brain and is not an intragenic miRNA. Biological systems analysis reveals that their target genes are linked to the process networks of protein folding, mitosis, and cell adhesion (Additional files 2 and 3). Their GO process is said to be positive regulation of smooth muscle cell chemotaxis (Additional file 4). Hsa-let-7f-5p was down-regulated, especially in spinal-onset patients. This finding combined with the finding of a negative correlation between this miRNA and ALSFRS-R bulbar paralysis score (less is worse) may indicate a link between this miRNA and amount of muscle volume present in a patient. The percentage of spinal-onset patients was high in the discovery cohort (81 %) which may have lead to not seeing a significant difference in qPCR.

Hsa-miR-4299 is transcribed from chr11, and is not an intragenic miRNA. An analysis of biological systems revealed that their target genes are linked to the process networks of cell adhesion, regulation of angiogenesis, and axonal guidance (Additional files 2 and 3). Their GO processes are regulation of synapse assembly, organization, structure and activity, positive regulation of dense core granule biogenesis and central nervous system neuron development (Additional file 3). Hsa-miR-4299 was significantly down-regulated in both microarray analysis and qRT-PCR. The down-regulation we saw contradicts an ALS patients’ FFPE (formalin-fixed paraffin embedded) sample study which revealed that within 364 miRNAs in the motor cortex, 91 were up-regulated and 233 were down- regulated, and hsa-miR-4299 was notably up-regulated [21]. The FFPE sample study used neural, glial and endothelial cells and is considered to be a reflection of intracellular miRNAs. Plasma miRNAs are secreted from the cell and circulate, hence, the contradictive expression of hsa-miR-4299 may be explained by the intra- or extracellular conditions. There are some possible explanations for this phenomenon; there is a compensatory reaction in the neural cells which modify ALS progress, or there is a pathological mechanism change in the secreting of miRNAs.

There are some commonly predicted target genes for some of the miRNAs, and according to miRmap, *EPHA4* is the most common and notable target gene of hsa-miR-4649-5p and hsa-miR-4299 (Additional file 2). *EPHA4* has been reported to be highly expressed in animal models and humans, and is said to be a disease modifier of ALS. Its expression inversely correlates with disease onset and survival, and its knockdown rescues the axonopathy induced by expression of mutant *TARDBP* and *SMN* [22]. Up-regulated hsa-miR-4649-5p and hsa-miR-4299 are predicted to down-regulate *EPHA4*, and is surmised as being a compensating mechanism for neuronal death. But, on the other hand, a dramatic down-regulation of hsa-miR-4299 in plasma could be a possible factor for deterioration. In the future, an intervention of miRNAs which regulate disease modifier genes could in turn, moderate the progress of ALS.

## Conclusion

We have shown the relationship circulating plasma miRNA has, with both disease patients and healthy controls. Hsa-miR-4649-5p showed up-regulation and hsa-miR-4299 showed down-regulation regardless of measurement methodology, and they have the potential to be ALS diagnosis biomarkers.

## Materials and methods

### Patients and participants

Sixteen sALS patients and ten healthy controls from Hokkaido University Hospital sampled Aug 2008 ~ Sep 2012 were included in the discovery cohort. For the validation cohort, 48 other sALS patients and 47 other healthy controls from Hokkaido University Hospital and Obihiro Kosei General Hospital sampled Oct 2009 ~ Sep 2013 were included. All sALS patients were diagnosed by board-certified neurologists as being at least clinically possible under the El Escorial and Airlie House diagnostic criteria [23]. Patients expressing only an upper or lower motor deficiency or presenting with coexisting neurological disorders were excluded. All participants did not have other serious diseases or infections of hepatitis B virus (HBV), hepatitis C virus (HCV), human immunodeficiency virus (HIV) or human T-cell leukemia virus type 1 (HTLV-I). The C9ORF72 mutation of familial ALS was excluded in all sALS patients. As part of the scale assessment, each of the sALS patient’s clinical demographics; age at time of collection, sex, disease duration from onset to collection and onset to end point, initial symptoms, ALSFRS-R [24], bulbar paralysis scores of ALSFRS-R, and Barthel Index were noted [25]. The patient’s date of death or start date of invasive mechanical ventilation was set as the end point of the observation. Further, for every traceable patient, we noted in detail the disease duration from onset to end point. We were able to collect and analyze twice for seven of the patients in the validation cohort.

We received consent forms from all participants in the study, and the study was approved by the Ethical Standards Committee of both institutions.

### Sample preparation and microarray analysis

Blood was collected in disodium ethylenediaminotetraacetate (Na2EDTA) tubes and centrifuged immediately, after which the plasma was separated and frozen at −80 °C. RNA (total RNA) was extracted from 300 μl of the frozen plasma using a 3D-Gene® RNA extraction reagent (TORAY Industries, Inc., Tokyo, Japan) and the half of the extracted product was used. After being labeled with an Exiqon miRCURY LNA™ microRNA Array Power Labeling kit (Exiqon Inc., Woburn, MA, USA), miRNAs were analyzed with a 3D-Gene® Human miRNA oligo chip (Ver. 17.0) (TORAY Industries, Inc., Tokyo, Japan) which carries approximately 1800 probes to detect human miRNAs using fluorescent signals. From the outputted signal data, we subtracted the background noise, and calibrated using the global normalization method with the median value as 25.

Comparisons of all normalized miRNA data calculated as a binary logarithm underwent a paired *t* test, Wilcoxon rank sum test, fold change ratio and variance using JMP® Pro 11.0.0. (SAS Institute Inc., Cary, North Carolina, USA). MiRNA showing the most significant changes either up-regulating or down-regulating in sALS patients, were chosen as the biomarker candidates for validation analysis.

### Validation of miRNAs with RT-qPCR

The total RNA was extracted from frozen plasma (200 μl) using a miRNeasy Serum/Plasma Kit (QIAGEN Inc., Valencia, CA, USA). C.elegans miR-39-3p (Cel-miR-39-3p) synthetic oligonucleotide RNA (Sigma-Aldrich, Saint Louis, Missouri, USA) (400amol), used as a spike-in control was added to the plasma after the addition of a denaturing solution.

The concentration of the extracted RNA was measured using Bioanalyzer 2100 with an Agilent RNA 6000 pico kit and Agilent small RNA kit (Agilent Technology Inc., Urdorf, Switzerland). For cDNA synthesis, 1 ng of total RNA was reverse calculated from the concentration and was reverse transcribed in a 20 μl reaction using a miScript II RT kit (QIAGEN Inc., Valencia, CA, USA).

A miScript SYBR® Green PCR Kit (QIAGEN Inc., Valencia, CA, USA) and miScript Primer Assays (QIAGEN Inc., Valencia, CA, USA) were used on a Applied Biosystems® StepOnePlus^TM^ real time PCR system (Applied Biosystems, Foster City, CA, USA) to quantify the plasma miRNAs. There were 11 Qiagen miScript primers used: hsa-miR-4258, hsa-miR-663b, hsa-miR-4649-5p, hsa-miR-26b-5p, hsa-miR-4299, hsa-let-7f-5p, hsa-miR-4419a, hsa-miR-3187-5p, hsa-miR-4496, cel-miR-39-3p and hsa-miR-4516. Hsa-miR-4516 was used as an internal control because it showed minimum variance and approximation value between the controls and patients in miRNA microarray analysis. Run in duplicate 25 μl reactions, comparative quantification was used to determine the relative quantities of miRNA from hsa-miR-4516. Each of the comparative quantification values, along with the clinical characteristics were analyzed with Student’s -*t* test, receiver operating characteristic curve and Pearson product–moment correlation coefficient using JMP® Pro 11.0.0. A P value less than 0.05 (*) was considered statistically significant.

## Additional files

Additional file 1:
**Calculated copy numbers of hsa-miR-663b showed increases over time.** (PDF 30 kb) Additional file 2:
**The most predictable target genes found by miRmap.** (CSV 1 kb) Additional file 3:
**Results of process network analysis from Metacore™.** (CSV 2 kb) Additional file 4:
**Results of Gene Ontology analysis from Metacore™.** (CSV 2 kb)

## Abbreviations

miRNA: microRNA
ALS: Amyotrophic lateral sclerosis
AGO2: Argonaute 2
SMA: Spinal muscular atrophy
CSF: Cerebrospinal fluid
AUC: Area under the curve
sALS: Sporadic amyotrophic lateral sclerosis
RT-qPCR: Reverse transcription-quantitative polymerase chain reaction
PD: Parkinson’s disease
ALSFRS-R: ALS functional rating scale revised
GO: Gene ontology
FFPE: Formalin-fixed paraffin embedded
Na2EDTA: Disodium ethylenediaminotetraacetate
HBV: Hepatitis B virus
HCV: Hepatitis C virus
HIV: Human immunodeficiency virus
HTLV-I: Human T-cell leukemia virus type 1
