# Supplementary figures and images for: Identification of plasma microRNAs as a biomarker of sporadic Amyotrophic Lateral Sclerosis
Source: Mol Brain. 2015 Oct 24;8:67. doi: 10.1186/s13041-015-0161-7 (PMC4619470; doi:10.1186/s13041-015-0161-7)

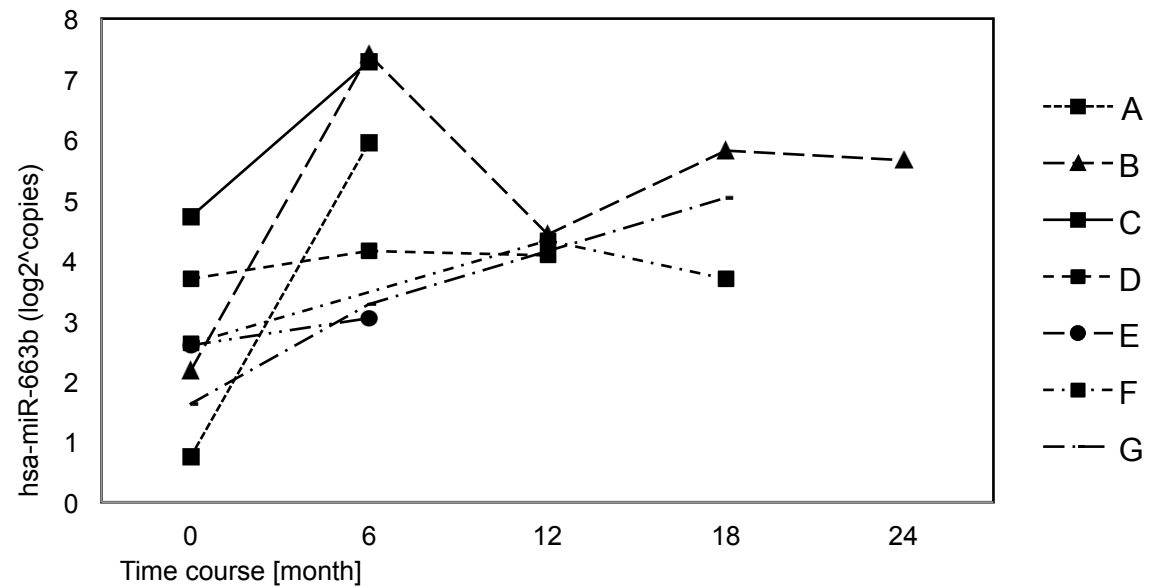

Supplement: Additional file 1: — Calculated copy numbers of hsa-miR-663b showed increases over time. (PDF 30 kb) [file 13041_2015_161_MOESM1_ESM.pdf]
